# Chromosome-level genome assembly of the small yellow croaker, *Larimichthys polyactis* (Perciformes: Sciaenidae)

**DOI:** 10.1038/s41597-025-05072-y

**Published:** 2025-04-30

**Authors:** Feng Liu, Wei Zhan, Dandan Guo, Ting Ye, Bao Lou

**Affiliations:** 1 Zhejiang Key Laboratory of Coastal Biological Germplasm Resources Conservation and Utilization, Institute of Hydrobiology, Zhejiang Academy of Agricultural Sciences, Hangzhou, 310021 China; 2 State Key Laboratory for the Quality and Safety of Agro-products, Zhejiang Academy of Agricultural Sciences, Hangzhou, 310021 China

**Keywords:** Ichthyology, Next-generation sequencing, Genome evolution, High-throughput screening

## Abstract

Small yellow croaker (*Larimichthys polyactis*) represents an important commercial fish species in China. A high-quality genome is essential for evaluating the fine-scale genetic structure, which has significant implications for the conservation of wild stocks, fishery management, and the utilization of germplasm in *L. polyactis*. This study presents a chromosome-level genome of *L. polyactis* generated by employing the PacBio high-fidelity (HiFi) and high-throughput chromosome conformation capture (Hi-C) technologies. The complete genome spans 677.35 Mb with a scaffold N50 size of 28.51 Mb. A substantial portion of the genome, totaling 663.13 Mb (97.90%), was anchored to 24 chromosomes. Based on Benchmarking Universal Single-Copy Ortholog (BUSCO) analysis, *L. polyactis* exhibits high genomic completeness (98.00%). A total of 28,640 annotated genes were identified, with 25,801 being functionally annotated. The comparisons of 24 chromosomes between *L. polyactis* and *L. crocea* proved high conservation of synteny between this pair of relatives. These findings provide valuable resources for the conservation, functional genomics, molecular breeding and evolutionary studies of *L. polyactis*.

## Background & Summary

As a commercially important sciaenid fish species, the small yellow croaker (*Larimichthys polyactis*) inhabits temperate coastal waters spanning the Bohai, Yellow, and East China Seas^1^. This species holds cultural significance in Chinese dietary traditions and has emerged as a key aquaculture species since the 2015 development of controlled reproduction techniques, with primary production concentrated in the Zhejiang-Jiangsu coastal aquaculture zones^2^.

As an economically valuable fishery resource and ecologically important marine species in China, *L. polyactis* suffers from inadequate genomic resources. Recent transcriptomic studies have identified genes that contribute to adaptations to temperature variations and low oxygen conditions in *L. polyactis*, providing insights into stress response mechanisms in eurythermic marine fishes^3,4^. While currently available genomic resources for *L. polyactis* comprise preliminary genetic maps^5,6^ and two genome assemblies (706.15 Mb with 1.21 Mb contig N50; 653.32 Mb, with 2.47 Mb contig N50)^7,8^, the lack of a chromosome-level reference genome with high accuracy poses substantial limitations for genome-scale genetic breeding, effective management of wild populations, and development of science-based stock replenishment protocols for sustainable aquaculture practices.

This study presents a chromosome-level genome assembly for *L. polyactis* generated through an integrative approach combining two technologies: (1) PacBio high-fidelity (HiFi) circular consensus sequencing (CCS) for highly accurate long reads, and (2) high-throughput chromosome conformation capture (Hi-C) chromatin interaction data for chromosome-scale scaffolding. This represents the most complete genome assembly currently available, marking an important advance in recent studies and facilitates germplasm resource conservation efforts while supporting its use in genetic breeding.

## Methods

### Ethics statement

The animal study was reviewed and approved by Committee of Laboratory Animal Experimentation at Zhejiang Academy of Agricultural Sciences (Approval Code: 2021ZAASLA26, Approval Data: 1 March 2021).

### Sample collection and genome sequencing

Muscle tissue and blood samples were collected from a gynogenesis-derived homozygous female *L. polyactis* cultured under controlled conditions at Xiangshan Gangwan Aquatic Seeds Co., Ltd. (Ningbo, Zhejiang, China). Genomic DNA was isolated using the E.Z.N.A. A Tissue DNA Kit (Omega Bio-Tek, Norcross, GA, USA) following the manufacturer’s protocol. DNA integrity was verified by 1% agarose gel electrophoresis showing clear high-molecular-weight bands, while quantification was performed using a Qubit Fluorometer (Invitrogen, CA, USA) with A260/A280 ratios between 1.8–2.0 indicating high purity. High-quality DNA was used for subsequent experiments. For short-read sequencing, a genomic library with 450 bp insert size was constructed according to the manufacturer’s standard protocol and sequenced in paired-end 150 bp (PE150) mode on a NovaSeq 6000 platform (Illumina, CA, USA), generating 89.14 Gb raw data (~130 × genome coverage). Long-read sequencing was performed using the SMRTbell Express Template Prep Kit (Pacific Biosciences, Menlo Park, CA, USA) on a PacBio Sequel II system, yielding 53.65 Gb HiFi reads with the longest length, and N50 of read length being 45,575, and 15,358 bp, respectively. To achieve chromosome-level scaffolding, a Hi-C library was prepared from blood samples following standard Hi-C library preparation protocols by Shanghai Biozeron Biotechnology Co., Ltd. (Shanghai, China) and sequenced on an the NovaSeq 6000 platform. Raw Hi-C data were filtered to obtain 78.71 Gb clean reads, with Q20 = 96.61% and Q30 = 90.97%, which was used to assist chromosome anchoring (Table 1).

**Table 1:** Summary of obtained sequencing data generated from multiple sequencing technologies for *L. polyactis* genome assembly.

	Clean data (Gb)	Read N50 (bp)	Contig N50 (bp)	GC content (%)	Q20 (%)	Q30 (%)
Illumina short reads	86.07	575,836,130	—	42.77	96.95	92.07
PacBio HiFi reads	53.65	3,610,502	45,575	—	—	—
Hi-C	73.64	510,318,974	—	42.79	96.61	90.97

### Genome assembly and quality assessment

**Fig. 1:**
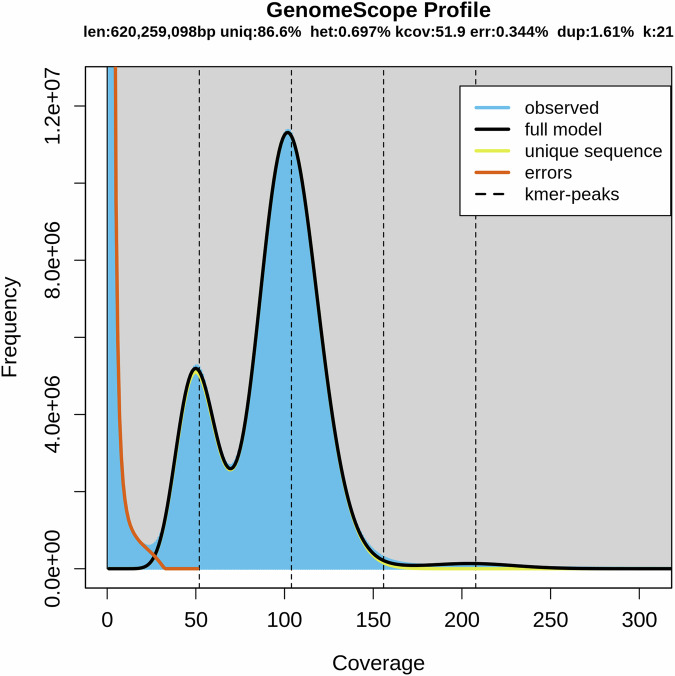
Estimated characteristics of the *L. polyactis* genome based on a 21-mer count histogram from Illumina short-read data. The estimated genome size was approximately 620,259,098 bp, with a heterozygosity rate of 0.697% and a repeat content of 13.4%.

The genome size, heterozygosity, and repetitive element content of *L. polyactis* were estimated through 21-mer frequency analysis (k = 21) using GCE (version 1.0.2)^9^, based on Illumina short-reads sequencing data. The analysis yielded an estimated genome size of 620,259,098 bp, with a low heterozygosity rate of 0.697% and moderate repeat content of 13.4% (Table 2; Fig. 1).

**Table 2:** Assembly features of the female *L. polyactis* genome.

Assembly feature	*L. polyactis*
K-mer	21
Genome size (Mb)	620,259,098
Heterozygous ratio (%)	0.697
Repeat (%)	13.4

**Fig. 2:**
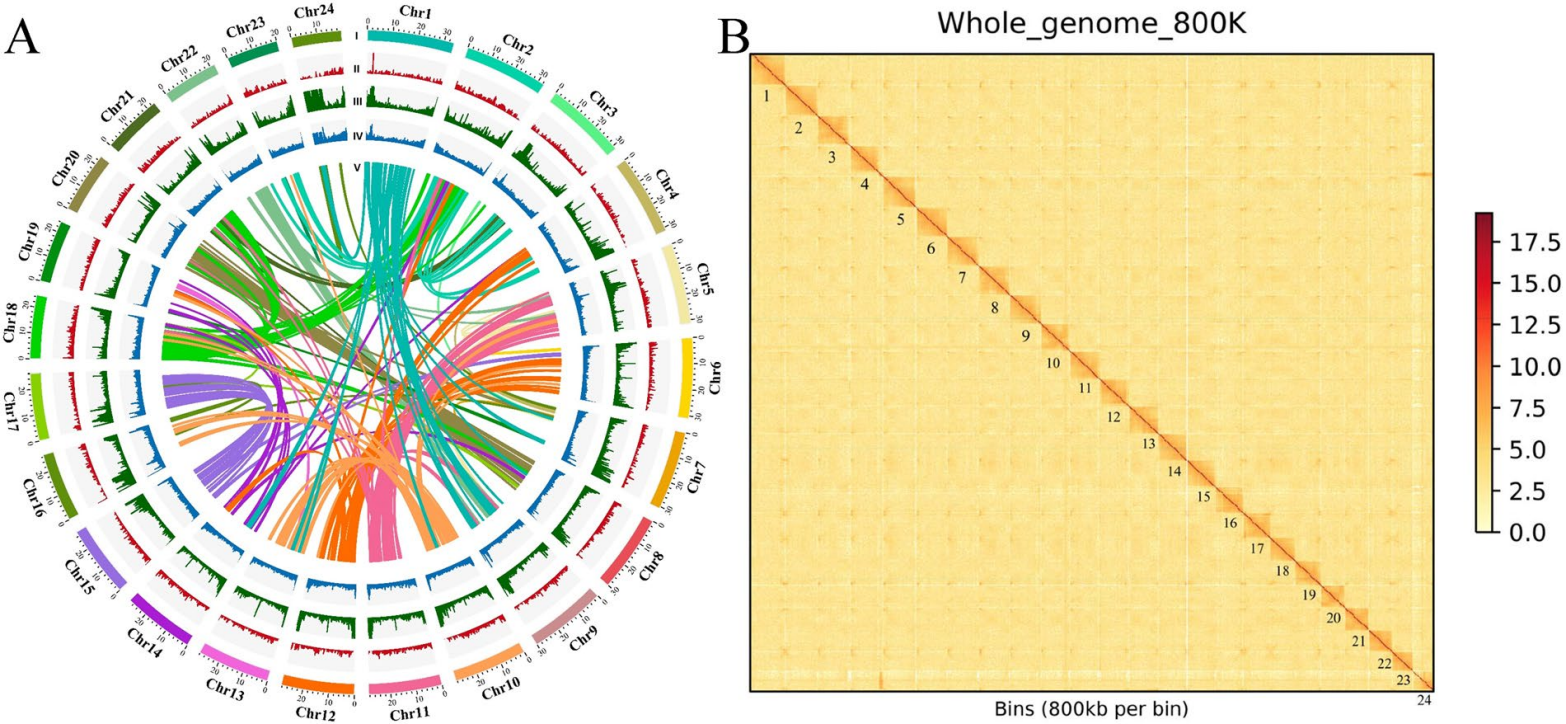
Characteristics of the *L. polyactis* genome. (**A**) Genomic landscape of *L. polyactis* genome. From outer to inner tracks: the chromosomes (Chr1 ~ Chr24) of *L. polyactis* genome; gene density across the genome; repeat sequence content; GC content across the genome; collinear gene blocks between chromosomes. Each linking line in the center of the circle connects pairs of homologous genes. (**B**) Genome-wide analysis of chromatin interactions at a 800 kb resolution in the assembled *L. polyactis* genome. Color blocks represent the interactions, with various strength from yellow (low) to red (high).

For *de novo* assembly, PacBio HiFi reads were processed via the pbcc pipeline with default parameters (https://github.com/PacificBiosciences/ccs), to generate circular consensus sequences. These sequences were subsequently assembled into contigs using hifiasm (version 0.16.1) (https://github.com/chhylp123/hifiasm) with default parameters. Hi-C data were integrated via ALLHIC (version 0.9.8; https://github.com/tanghaibao/allhic) to scaffold contigs into pseudochromosomes. The final chromosome-level assembly comprised 24 pseudochromosomes (total length: 677.35 Mb; contig N50: 25.99 Mb), with 97.89% (663.13 Mb) anchored to chromosomes and a Chr N50 of 28.51 Mb (Table 3; Fig. 2). Chromosome lengths ranged from 19.65 Mb to 33.83 Mb (Table 4). This assembly demonstrates improved quality compared to previous *L. polyactis* genomes reported by Xie *et al*.^7^ (total length: 706.15 Mb; contig N50: 1.21 Mb) and Wang *et al*.^8^ (total length: 653.32 Mb; contig N50: 2.47 Mb), with a higher chromosomal anchoring rate (97.89%) than other Sciaenidae species, such as *L. crocea* (92.48%)^10^, *Nibea albiflora* (92.20%)^11^, and *C. lucidus* (96.86%)^12^.

**Table 3:** Statistics of the *L. polyactis* genome assembly.

Content	Values	
	scaffold	contig
Assembly size (bp)	677,353,566	/
Longest scaffold /contigs (bp)	33,834,664	32,393,602
Number of scaffolds /contigs	24	185
N50 Scaffold/ Contig length (bp)	28,510,860	25,988,828
N90 Scaffold/ Contig length (bp)	23,429,296	5,575,771

**Table 4:** Detailed results of chromosome-level scaffolding via Hi-C technology.

Chromosome	Length (bp)	Chromosome	Length (bp)
Chr1	33,834,664	Chr13	28,232,251
Chr2	32,557,454	Chr14	27,992,954
Chr3	31,487,815	Chr15	27,795,422
Chr4	31,480,392	Chr16	26,811,182
Chr5	31,404,282	Chr17	26,169,382
Chr6	31,080,579	Chr18	24,678,019
Chr7	30,749,753	Chr19	24,244,764
Chr8	30,083,533	Chr20	23,779,637
Chr9	30,105,284	Chr21	23,429,296
Chr10	28,544,608	Chr22	21,913,216
Chr11	28,510,860	Chr23	20,151,093
Chr12	28,437,554	Chr24	19,651,708
Linked length (bp)	663,125,702		
Total length (bp)	677,353,566		
Linked percent (%)	97.89		

Additionally, the genome completeness was assessed using benchmarking universal single-copy orthologs (BUSCO) software (version 3.0.2)^13^, revealing 4,492 (98.0%) complete BUSCOs and 31 (0.7%) fragmented BUSCOs (Table 5). These values exceed those of the previously published *L. polyactis* genome (93.9% completeness), confirming the high quality of the current assembly for downstream genomic studies.

**Table 5:** Assessment of *L. polyactis* genome completeness by BUSCO.

Type	Gene number	Percent (%)
Complete BUSCOs (C)	4492	98%
Complete and single-copy BUSCOs (S)	4400	96%
Complete and duplicated BUSCOs (D)	92	2%
Fragmented BUSCOs (F)	31	0.7%
Missing BUSCOs (M)	61	1.3%
BUSCO evaluation	4584	100

### Functional annotation and evolutionary analysis

Repetitive sequences were annotated using Tandem repeats finder (version 4.07b)^14^, RepeatMasker and RepeatProteinMask (version 4.0.7, http://www.repeatmasker.org/) with the Repbase library^15^. *De novo* repeats were predicted via RepeatModeler (version 1.0.5, http://www.repeatmasker.org/RepeatModeler/), and a non-redundant repeat library was generated by integrating known, novel, and tandem repeats. Protein-coding genes were predicted using a dual approach: (1) *de novo* prediction with AUGUSTUS (version 3.2.3)^16^ on the repeat-masked genome, and (2) homology-based prediction.

As a result, a total of 34.55% of the genome was annotated as repetitive elements (Table 6). A total of 28,640 protein-coding genes were identified, with an average gene length of 1,632 bp (Table 7). Among these genes, 25,801 (90.09%) had homology with known genes in public databases, whereas 11,081 genes (38.69%) were annotated in all databases (Table 8). Additionally, 11,594 noncoding RNAs were annotated in the *L. polyactis* genome, with a total length of 1,008,174 bp (0.15% of the whole-genome length). These included 1,138 microRNAs, 238 rRNAs, 8,714 tRNAs, and 1,503 snRNAs (Table 9).

**Table 6:** General statistics of repetitive sequences in *Larimichthys polyactis* genome.

Type	Number	Repeat Size	% of genome
Tandem repeats	315,637	62,520,663	9.23
Interspersed repeat	1,237,716	171,527,318	25.32
LTR	129,364	19,202,743	2.84
DNA	503,372	53,698,728	7.93
LINE	138,151	35,935,792	5.31
SINE	26,927	3,415,792	0.50
RC	32,198	8,813,571	1.30
Unknown	407,704	64,038,519	9.45
Total	1,553,353	234,047,981	34.55

Note: Some elements may partly include another element domain.

**Table 7:** General statistics of the predicted protein-coding genes.

Genome Size (bp)	Gene Number	Gene Total Length (bp)	Gene Average Length (bp)	% of Genome (Genes)	Intergenic region Length (bp)	% of Genome (Intergenic)
677,353,566	28,640	46,751,496	1,632	6.90	630,602,070	93.10

**Table 8:** Functional annotation of the predicted genes in the assembly of *Larimichthys polyactis*.

Type	Gene number	Percentage (%)
Total	28,640	100
Nr	25,778	90.01
Swissprot	22,149	77.34
KEGG	15,765	55.05
COG	18,632	65.06
GO	17,658	61.66
Annotated in at least one database	25,801	90.09
Annotated in all database	11,081	38.69
Unannotated	2,839	9.91

**Table 9:** Non-coding RNAs in the *L. polyactis* assembly.

Type	Number	Average length (bp)	Total length (bp)	% of genome
tRNA	8,714	75	658,455	0.0972
rRNA_5S	121	117.7	14,247	0.0021
rRNA_5.8S	117	154	18,020	0.0027
sRNA	1	58	58	0
snRNA	1,503	149	224,266	0.0331
miRNA	1,138	81	93,128	0.0137
Total	11,594		1,008,174	0.1488

Note: “% of genome” was calculated by the non-gap genome size 677,353,566 bp.

**Fig. 3:**
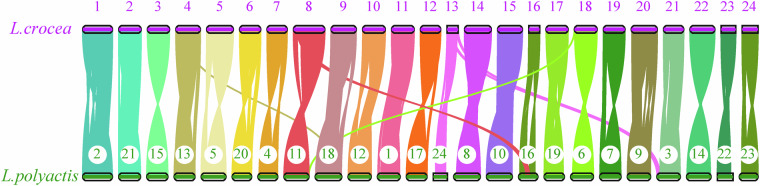
Genome synteny between *L. polyactis* and *L. crocea*. The results showed high synteny between *L. polyactis* and *L. crocea*, with one-to-one chromosomal correspondence. Minor rearrangements were observed in chromosomes 3, 11, 16, and 18, further validating the assembly’s completeness.

Chromosomal collinearity analysis (MCScanX; BLAST E ≤ 1e-10, 5 hits) revealed high synteny between *L. polyactis* and *L. crocea*, with one-to-one chromosomal correspondence (Fig. 3). Minor rearrangements were observed in chromosomes 3, 11, 16, and 18, further validating the assembly’s completeness.

## Data Records

The genomic project of *L. polyactis* has been uploaded to NCBI. The datasets for Illumina, PacBio HiFi, and Hi-C are accessible using the identifiers SRR32174815^17^, SRR32748863^18^ and SRR32162007^19^. The assembled genome has been submitted to the NCBI database under the accession number GCA_040670005.1^20^. The annotation results have been uploaded in figshare^21^.

## Technical Validation

DNA quality assessment was performed for both sequencing platforms. Electrophoretic analysis revealed high-molecular-weight DNA fragments (>30 kb) suitable for PacBio sequencing. Nucleic acid purity was verified by spectrophotometry (NanoDrop ND-1000, LabTech) with A260/A280 ratios meeting high-quality DNA standards. For Illumina sequencing, DNA concentrations quantified by fluorimeter (Qubit 4.0, Thermo Fisher) exceeded minimum requirements for library construction. Genome characterization included two key analyses: (1) 21-mer frequency distribution estimation using Illumina short reads to predict genome size, and (2) assembly evaluation with BUSCO (v5) showing >98% complete ortholog recovery. Comparative genomics revealed 24 conserved syntenic blocks between L. polyactis and L. crocea, validating the chromosomal-level accuracy of our assembly.

## Supplementary information

Supplementary tables

## Data Availability

No custom code was used during this study for the curation and/or validation of the dataset. All instructions and sequences of actions carried out during data processing were performed in accordance with the guidelines and procedures outlined in the manual and protocols of the relevant bioinformatics software. If no detail parameters were mentioned for the software, default parameters were used as suggested by developer.
